# Novel Pharmacologic Targeting of Tight Junctions and Focal Adhesions in Prostate Cancer Cells

**DOI:** 10.1371/journal.pone.0086238

**Published:** 2014-01-31

**Authors:** Patrick J. Hensley, Andreas Desiniotis, Chi Wang, Arnold Stromberg, Ching-Shih Chen, Natasha Kyprianou

**Affiliations:** 1 Department of Urology, University of Kentucky College of Medicine, Lexington, Kentucky, United States of America; 2 Department of Pathology, University of Kentucky College of Medicine, Lexington, Kentucky, United States of America; 3 Department of Biostatistics, University of Kentucky College of Public Health, Lexington, Kentucky, United States of America; 4 Department of Statistics, University of Kentucky, Lexington, Kentucky, United States of America; 5 Division of Medicinal Chemistry, Ohio State University College of Pharmacy, Columbus, Ohio, United States of America; 6 Department of Toxicology, University of Kentucky College of Medicine, Lexington, Kentucky, United States of America; 7 Department of Molecular & Cellular Biochemistry, University of Kentucky College of Medicine, Lexington, Kentucky, United States of America; Thomas Jefferson University, United States of America

## Abstract

Cancer cell resistance to anoikis driven by aberrant signaling sustained by the tumor microenvironment confers high invasive potential and therapeutic resistance. We recently generated a novel lead quinazoline-based Doxazosin® derivative, DZ-50, which impairs tumor growth and metastasis via anoikis. Genome-wide analysis in the human prostate cancer cell line DU-145 identified primary downregulated targets of DZ-50, including genes involved in focal adhesion integrity (fibronectin, integrin-α6 and talin), tight junction formation (claudin-11) as well as insulin growth factor binding protein 3 (IGFBP-3) and the angiogenesis modulator thrombospondin 1 (TSP-1). Confocal microscopy demonstrated structural disruption of both focal adhesions and tight junctions by the downregulation of these gene targets, resulting in decreased cell survival, migration and adhesion to extracellular matrix (ECM) components in two androgen-independent human prostate cancer cell lines, PC-3 and DU-145. Stabilization of cell-ECM interactions by overexpression of talin-1 and/or exposing cells to a fibronectin-rich environment mitigated the effect of DZ-50. Loss of expression of the intracellular focal adhesion signaling effectors talin-1 and integrin linked kinase (ILK) sensitized human prostate cancer to anoikis. Our findings suggest that DZ-50 exerts its antitumor effect by targeting the key functional intercellular interactions, focal adhesions and tight junctions, supporting the therapeutic significance of this agent for the treatment of advanced prostate cancer.

## Introduction

Prostate cancer is the second most common cancer among men, with 206,640 men diagnosed and 28,088 dying from prostate cancer in 2012 [1]. Chemotherapeutic targeting of the androgen signaling axis in prostate cancer has contributed to the best cancer survival rate in men. However, a subset of patients become refractory to androgen ablation therapy by failing apoptosis and progressing to castration resistant prostate cancer (CRPC) [2]. Prostatic glandular epithelial cells have an intrinsic need for survival signals imparted by intercellular and cell-extracellular matrix (ECM) interactions. Focal adhesions are vital for both normal contact-dependent signaling by normal cells and invasion, migration and metastasis of malignant cells.

Work from this laboratory identified new anti-tumor action exerted by drugs classically used for the treatment of benign prostatic hyperplasia (quinazoline-α_1-_adrenoceptor antagonists) via induction of the extrinsic apoptosis cascade (death receptor activation, caspase-8 cleavage and inhibition of AKT survival signaling) [3], [4], [5]. Structural optimization led to the generation of novel compounds capable of anoikis induction and inhibition of angiogenesis [6], [7]. Anoikis, apoptotic cell death consequential to insufficient cell-ECM interactions, is a critical component of angiogenesis and metastasis [8]. Aggressive tumor cells subvert this mechanism, maintaining survival through dissemination and seeding in distant organs [9]. Anoikis resistance is closely linked to increased metastatic potential in many human malignancies, including prostate cancer [10], renal cell carcinoma [11], breast cancer [12] as well as tumors of mesenchymal origin [13].

Metastasis necessitates disruption of cellular interactions with the tumor microenvironment, increased migratory and invasion capacity and the ability to overcome the pro-apoptotic signals imparted by diminished intercellular and cell-ECM interactions [14]. The ECM comprises a diverse network of cytokines impacting cell growth, motility and angiogenesis which can be made available for cellular use by enzymatic digestion and remodeling [15]. Transmembrane integrins are characterized by bidirectional signaling. Oligomerization of integrin proteins about an ECM substrate induces conformational changes which are transmitted through the plasma membrane to modulate affinity of intracellular signaling effectors on cytosolic integrin tails [16]. The protein aggregates, collectively known as the focal adhesion complex (FAC), include actin binding proteins (talin-1, vinculin, vimentin, paxillin, filamin, ect.) which stabilize the cytoskeleton and kinases (focal adhesion kinase [FAK], integrin-linked kinase [ILK], and SRC non-receptor tyrosine kinase) which propagate intracellular signaling to the nucleus. This integrin-mediated “outside-in” signaling cascade controls processes vital to cellular function and growth as cell cycle progression and differentiation [17].

As the actin cytoskeletal network undergoes dynamic remodeling/organization, the integrin clustering induces “inside-out” signaling to increase affinity of integrins to the ECM, effectively establishing a focal adhesion [18]. Upon insufficient integrin-ECM interactions, cells downregulate members of the anti-apoptotic Bcl-2 family and upregulate Fas ligand (FasL), inducing anoikis via the extrinsic apoptosis pathway [19], [20]. Talin-1 functionally contributes to anoikis-resistance and prostate cancer metastasis by enhancing focal adhesion formation and Akt-survival signaling. We recently demonstrated a significant correlation between talin-1 overexpression and metastasis in a mouse model of prostate tumorigenesis and in human prostate cancer progression [10].

Pharmacological exploitation of the α1-adrenoreceptor antagonist doxazosin® has led to the generation of novel quinazoline-based compounds, with the lead agent, DZ-50, having potent anoikis-inducing effects against cancer cells [6]. DZ-50 suppresses growth of human prostate cancer xenografts and inhibits their metastatic potential *in vivo* by impairing angiogenesis, migration and invasion [7] through targeting the focal adhesion signaling axis [11]. The present study investigated the cellular targets of DZ-50 in androgen-independent human prostate cancer cells. Genome-wide analysis identified critical effectors of focal adhesion and tight junction interactions which are targeted by the novel quinazoline agent.

## Materials and Methods

### Cell Lines and Reagents

The androgen-independent human prostate cancer cell lines PC-3 and DU-145 were obtained from the American Type Tissue Culture Collection and cultured in RPMI 1640 (Invitrogen) containing 10% fetal bovine serum (Invitrogen) and antibiotics (PenicilinG/Streptomycin, 50 µg/mL). DU-145 cells were transfected with pEGFP or talin-1 plasmids and cloned under G418 selection (Life Technologies Bethesda Research Laboratories). For silencing talin-1 expression, the shRNA talin-1 vector (GIPZ shRNAmir talin-1) was used from Open Biosystems (Hunsville, AL) and shRNA talin1 DU-145 prostate cancer cells were selected under puromycin. Polyclonal populations were pooled under selection, and stable cell lines were characterized by immunoblotting. PC-3 cells were stably transfected with pTRIPZ vector containing TRE-ILK shRNA (Thermo Scientific). PC-3 shILK cells were induced with 2 µg/ml doxycycline (Enzo Life Sciences) for two days. DZ-50, a first-generation doxazosin derivative (Shaw et al., 2004; Garrison et al., 2007), was used at a concentration of 5 µM dissolved in DMSO for all treatments. Sterile DMSO was used for control/untreated samples.

### Cell Viability Assay

Cell viability was assessed after treatment with 5 µM DZ-50 using the colorimetric MTT (3-[4,5-dimethylthiazol- 2-yl]-2,5 diphenyltetrazolium) assay (1 mg/ml MTT in PBS), and quantified using a spectrophotometric measurement. Statistical analysis of 3 independent experiments, each performed in triplicate, is expressed relative to the untreated control.

### Cell Migration Assay

Wounding was inflicted using a sterile pipette tip in confluent cell monolayers in 6-well plates. After incubation for 12–48 hrs in the presence of DZ-50 (5 µM), wounded areas were examined by light microscopy (Axiovert 10, Zeiss). Cells migrating to the wounded areas were counted under a microscope. Migration potential was determined as the average number of cells in three random high-power (400×) fields/well. Numerical data are obtained from three independent experiments performed in triplicate and is expressed relative to untreated controls.

### Cell Adhesion Assay

Cultures of PC-3 and DU-145 sublines were treated (24 hrs) with DZ-50 (5 µM) and harvested. Cells (1×10^5^/well) were added to 6-well plates coated with fibronectin (5 µg/ml, BD Biosciences) and following a 30-mins incubation at 37°C, cells were fixed with 100% (v/v) cold methanol and subjected to image analysis. The number of adhered cells was counted in three representative fields/well (400×). Numerical data represent the average of three independent experiments performed in triplicate and are expressed relative to untreated controls.

### Western Blot Analysis

Human prostate cancer cells, DU-145 and PC-3, were treated with DZ-50 (5 µM) for sequential time periods and cell lysates were generated in lysis buffer (150 mmol/L NaCl, 50 mmol/L Tris (pH 8.0), 0.5% deoxycholic acid, 1% NP40 with 1 mmol/L phenyl methyl-sulfonyl fluoride, pH 7.4). Protein samples were subjected to SDS-PAGE and transferred onto Hybond-C membranes (Amersham Pharmacia Biotechnology). Membranes were incubated overnight at 4°C with the specific primary antibodies: Akt (Cell Signaling Technology), phosphorylated Akt Ser 473 (Cell Signaling Technology), GSK-3β (Cell Signaling Technology), phosphorylated GSK Ser 9 (Cell Signaling Technology), ILK-1 (Cell Signaling Technology), ZO-1 (Invitrogen), Claudin-11 (Santa Cruz Biotechnology), Talin-1 (Millipore) or Actin (Calbiochem). Following incubation with the respective primary antibody, membranes were exposed to horseradish peroxidase–labeled secondary antibodies and signal was detected with SuperSignal West Dura Extended Duration Substrate (Pierce) and exposed on X-ray film. Densitometric analysis was performed using ImageJ software and values are expressed relative to controls.

### Gene (qRT-PCR) Analysis

RNA was isolated from cell lysates using TRIzol Reagent (Ambion) and Pure Link RNA Mini Kit (Invitrogen) according to the manufacturer. After homogenization and phase separation by centrifugation (12,000 g, 4°C), RNA was precipitated with isopropanol. Samples were centrifuged (12,000 g, 4°C) and cDNA was synthesized using RNA (1 µg) and the Reverse Transcription System (Promega). DNA array analysis was conducted at the University of Kentucky Microarray Core Facility using Affymetrix GeneChip Technology. The transcripts were evaluated by ABI 7700 Sequence Detection System (Applied Biosystems Inc.); each treatment (5 µM DZ-50, 9 hrs) was performed in triplicate.

For the RT-PCR analysis, RNA (1 µg) was subjected to reverse transcription using the Reverse Transcription System (Promega). The following primers were designed (Sigma) for the SYBR Green quantitative real time PCR (qRT-PCR) system:Fibronectin-1 (F: 5′- TCATGAGGCAACGTGTTATGATG-3′, R: 5′- CGAGATATTCCTTCTGCCACTGT-3′),TALIN-1,(F:5′- GCAGAAGGGAGAGCGTAAGATC-3′, R: 5′-TGAGAGAACGGGCTAGCTTCA-3′), Integrin-α6 (F:5′-CAGAAAGTGTGCATGGAGGAAA-3′, R:5′- TGGGAATGGGACGCAGTT-3′), and ZO-1 (F: 5′- GGAGCTGCGCTTACCACACT-3′, R: 5′- TTTGCTCCAACGAGATAATTTGG-3′). The following primers were obtained for the TaqMan qRT-PCR system (Invitrogen): 18 s ribosomal RNA (rRNA), Thrombospondin-1, IGF-BP3, Claudin-11, Snail. cDNA was used for qRT-PCR analysis according to respective SYBR Green and TaqMan protocols (Bio-Rad). For the qRT-PCR experiments, each sample was analyzed in triplicates and data represent average values from three independent experiments. Numerical data for transcript levels were normalized to 18 s rRNA in controls and expressed relative to untreated controls.

### Immunofluorescence Analysis

Cells plated in 4-well chamber slides coated with Fibronectin (5 µg/ml, BD Biosciences) were exposed to DZ-50 treatment (5 µM) for 12 hrs. Cells were then fixed in 100% (v/v) methanol, and after blocking at 4°C (5% NGS, 0.3% Triton X), were exposed to the primary antibody (4°C, overnight). The following specific antibodies were used: ILK-1 (Cell Signaling Technology), ZO-1 (Invitrogen), Claudin-11 (Santa Cruz Biotechnology), Snail (Cell Signaling Technology), Talin (Millipore). Cells were then incubated with fluorochrome-conjugated secondary antibody (Invitrogen) (2 hrs, room temperature) and subjected to confocal microscopy using an Olympus FV1000 Confocal Microscope v1.21. and software version FV10-ASW 3.1.

### Microarray Analysis

shTalin or vector DU-145 human prostate cancer cells were treated with DZ-50. RNA samples from cells before or after (9 hrs) treatment were submitted to the University of Kentucky Microarray core facility for analysis on Affymetrix Human Gene 1.0 ST arrays (Affymetrix, Santa Clara, CA). The experiment under each condition was performed in duplicate.

### Statistical Analysis

Microarray data were normalized by using RMA and analyzed by using two-way analysis of variance (ANOVA) models, with genotype (shTalin or vector) and treatment (before or after) as the two factors. Contrasts were generated to evaluate changes in mRNA expression between treated versus untreated in vector cells. False discovery rate (FDR) and the associated q-values were calculated by using the method in REF. Differentially expressed genes were determined based on FDR<20% and fold change>1.5. Statistical analyses for data from all other experiments were performed based on one-sample or two-sample t-tests as appropriate. At P<0.05 values were considered statistically significant.

## Results

### Novel Quinazoline DZ-50 Induces Prostate Cancer Cell Anoikis

**Figure 1:**
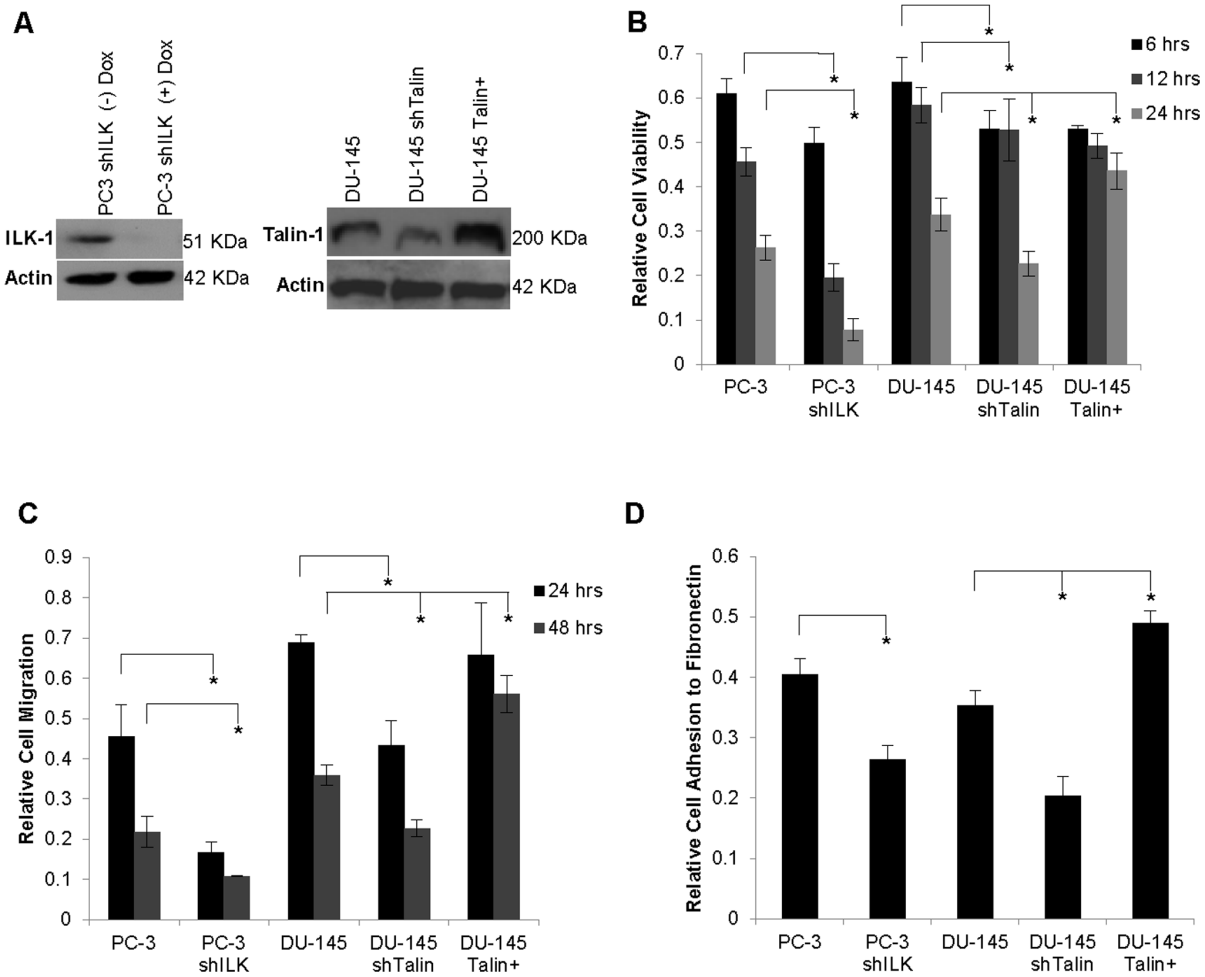
ILK and talin mediate DZ-50 action on prostate cancer cell death and migration. Panel A, right, Western blot revealing that shILK transfectant PC-3 cells exhibit total loss of ILK-1 protein expression relative to vector control cells upon induction with doxycycline (48 hrs). On the left, DU-145 cells in which talin has been silence (shTalin) or overexpressed (Talin+), exhibit significant reduction or marked overexpression of talin protein levels respectively, relative to parental DU-145 cells. Panel B, DZ-50 treatment leads to a significant decrease in prostate cancer cell viability in a time-dependent fashion. Loss of ILK1 enhances the DZ-50 induced loss of cell viability; in contrast talin overexpression confers resistance to DZ-50 induced cell death. Panel C, DZ-50 significantly reduces prostate cancer cell migration. Loss of talin results in a significant reduction of prostate cancer cell migration compared to parental control DU-145 cells. In response to DZ-50, talin overexpression restores cell migratory ability to the levels of untreated cells. Panel D, Functional loss of ILK in PC-3 prostate cancer cells and loss talin in DU-145 cells significantly impairs the ability of the respective prostate cancer cells to adhere to fibronectin (ECM). Talin overexpression markedly enhances prostate cancer cell adhesion to fibronectin, compared to parental DU-145 and DU-145 shTalin cells. Statistical significance was set at *p<0.05.

The anoikis-inducing effect of the lead quanazoline compound DZ-50 was investigated in human prostate cancer cell lines variably expressing the FAC proteins, talin-1 and ILK. Stable transfection of DU-145 cells resulted in knockdown of talin (DU-145 shTalin) and overexpression of talin (DU-145 Talin+) (Fig.1, panel A). PC-3 cells expressing an inducible shILK vector demonstrated effective downregulation of ILK upon induction with doxycycline for 48 hrs (Fig.1, panel A). There was a time-dependent decrease in cell viability in response to DZ-50 (Fig.1, panel B). Loss of ILK and talin expression in PC-3 and DU-145 prostate cancer cells respectively resulted in enhanced sensitivity to DZ-50, while talin overexpression suppressed anoikis. The data on panel C (Fig. 1) show that in response to DZ-50, prostate cancer ell migration is significantly increased. Loss of either ILK (PC-3 cells), or talin (DU-145 cells), inhibited cell migration, while further enhanced the effect of DZ-50. Loss of either ILK or talin independently led to a significant reduction in cell adhesion to fibronectin (Fig.1, panel D).

### Identification of Molecular Targets of Lead Quinazoline

**Figure 2:**
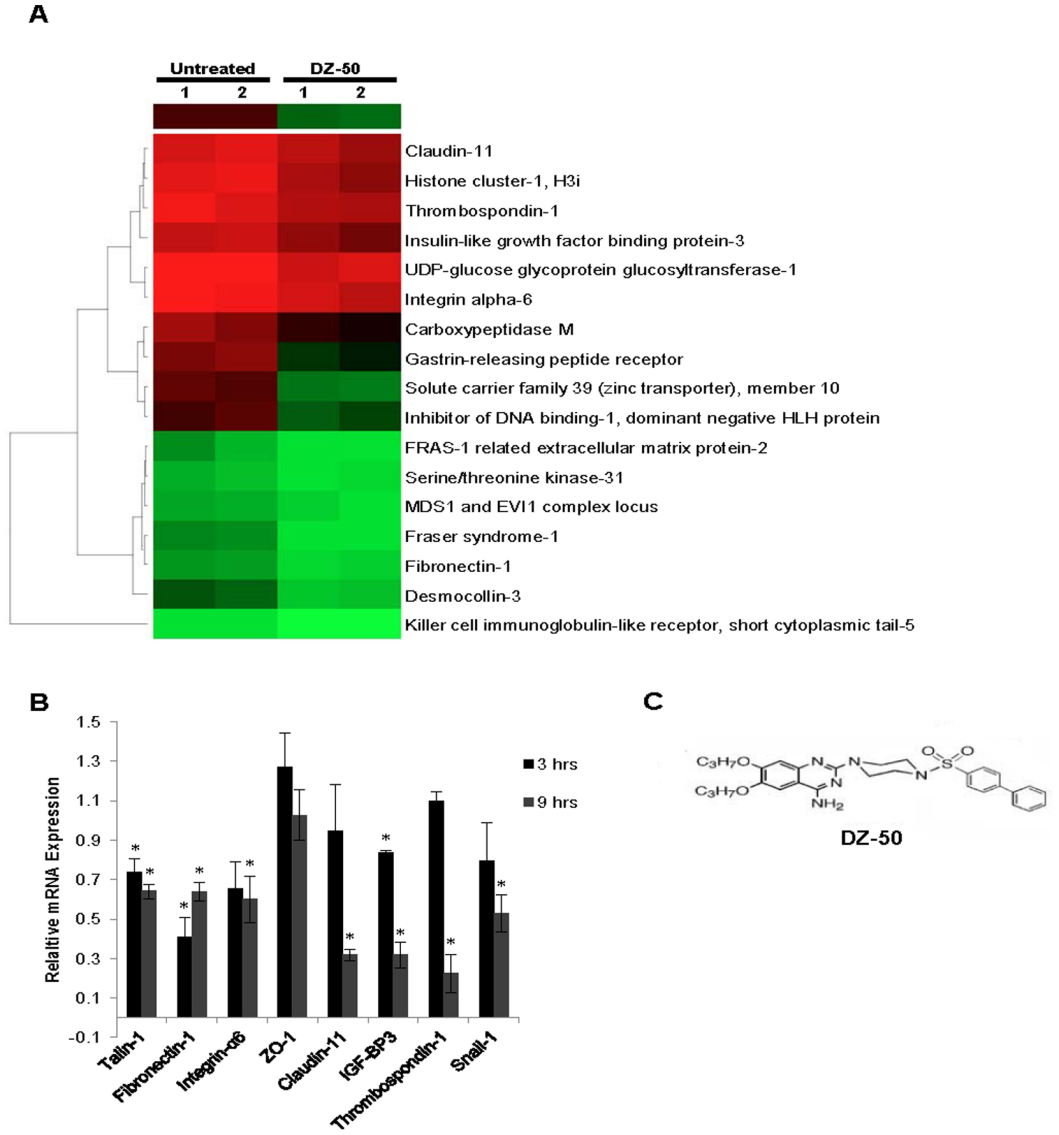
Genes targeted by DZ-50 in prostate cancer cells. Panel A, Heat map of differentially expressed genes in DU-145 human prostate cancer cells before and after treatment with DZ-50 (9 hrs). We identified 17 markedly downregulated genes following treatment with DZ-50 (9 hrs), including genes encoding for ECM regulators *fibronectin* and *integrin α6*, tight junction mediator *Claudin-11* and angiogenesis signaling effector *thrombospondin 1*. (fold change >1.5, false discovery rate <20%). Panel B, Validation of gene expression using qRT-PCR after DZ-50 treatment of prostate cancer cells (5 µM) for 3 and 9 hrs. A significant reduction in mRNA relative to untreated cells was detected for genes involved in ECM-focal adhesion signaling components *(fibronectin*, *integrin-α6* and *talin*), tight junctions (*claudin-11*), angiogenesis (*thrombospondin-1*) and EMT (*Snail*). *indicates significant difference at p<0.05. Panel C, Molecular structure of the Doxazosin© derivative, DZ-50.

Genome-wide analysis of gene expression in the human prostate cancer cell line DU-145 was used to identify primary targets of DZ-50 (Fig. 2). The heat map from the gene array analysis after treatment of prostate cancer cells with DZ-50 (for 9 hrs) is shown on Figure 2 (panel A). DZ-50 resulted in a significant downregulation of genes encoding plasma membrane associated proteins, including regulators of ECM (*fibronectin* and *integrin-α_6_*), and tight junctions (*claudin-11*), insulin growth factor binding protein 3 (*IGFBP-3*), as well as the angiogenesis mediator thrombospondin 1 (*TSP-1*). To validate the panel of candidate gene targets identified by the gene array molecular profiling, we subsequently conducted quantitative real time PCR (qRT-PCR) analysis (Fig. 2, panel B). Exposure of DU-145 cells to DZ-50 (3 and 9 hrs) led to a significant inhibition of expression for a selected gene signature identified the array analysis (Fig.2, panel A), including genes encoding for key focal adhesion signaling effectors, intracellularly (talin) and extracellularly (fibronectin) and tight junction proteins (Claudin-11 and ZO-1). A transcriptional mediator of EMT, *Snail,* is also reduced by DZ-50. The chemical structure of the quinazoline-based compound DZ-50, is shown on panel C (Fig. 2).

### DZ-50 Targets Vital Cellular Interactions in Prostate Cancer Cells

**Figure 3:**
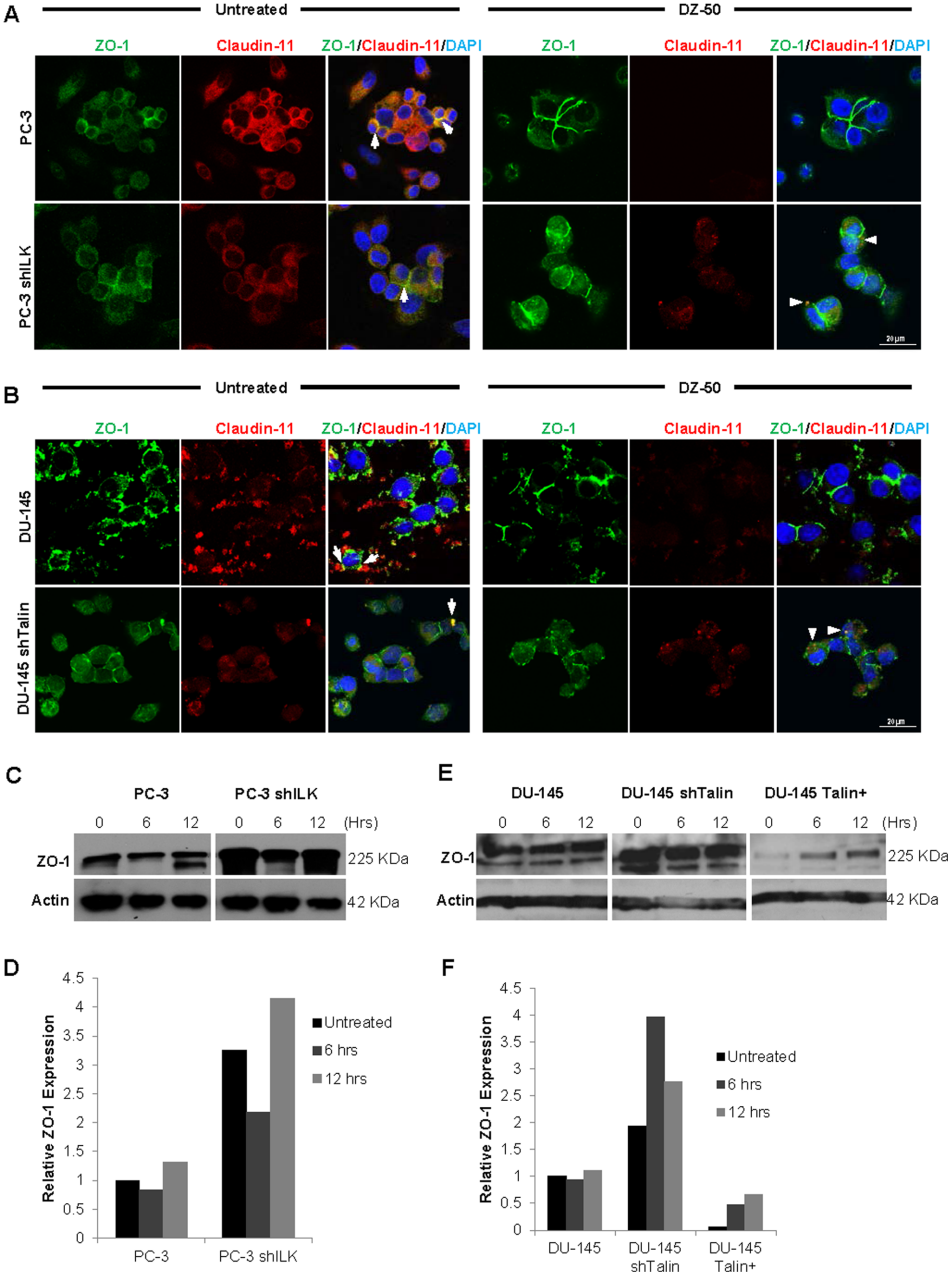
Disruption of tight junctions in human prostate cancer cells by lead agent DZ-50. Characteristic confocal microscopy images of PC-3 cells (Panel A) and DU-145 cells (Panel B). Treatment with DZ-50 (12 hrs, 5 µM) decreases Claudin-11 expression and inhibits tight junction formation. Tight junctions complexes (arrows), characterized by colocalization of the tight junction proteins Claudin-11 (red) and ZO-1) (green) is completely abrogated by DZ-50. In PC-3 shILK and DU-145shTalin cells there is weak formation of TJ complexes (arrow heads), in response to DZ-50. DAPI (blue) is used for nuclear detection (Panels A and B). Magnification x100. Panels C–F**,** Western blots and respective densitometric analysis revealing the expression of TJ protein ZO-1 in response to DZ-50 in PC-3 (Panels C and D) and DU-145 (Panels E and F) prostate cancer cell lines.

To characterize the effect of DZ-50 on tight junctions (TJ) the co-localization of ZO-1 and Claudin-11, two proteins essential for these intercellular interactions, was assessed using fluorescent confocal microscopy in two different human prostate cancer cell lines, PC-3 and DU-145 (Fig. 3, panels A and B respectively). Treatment with DZ-50 (12 hours) markedly decreased Claudin-11 expression (red) in both parental cell lines, resulting in appreciable impairment of TJ formation. Subcellular localization of ZO-1 (green) to the plasma membrane resulted in response to DZ-50. Cells expressing functional loss of the focal adhesion proteins ILK (PC-3 shILK) and talin (DU-145 shTalin) maintained some Claudin-11 expression despite treatment with DZ-50 relative to the respective parental cell lines (white arrows). This expression of Claudin-11 complexes with ZO-1 at the plasma membrane as poorly defined, punctate TJ complexes is shown on Figure 3 (composite images, panels A and B). In response to DZ-50, there was a time-dependent upregulation in ZO-1 protein levels. ZO-1 expression was inversely correlated with expression of focal adhesion protein ILK (Fig. 3, panels C and D). Western blot analysis revealed that talin overexpression in DU-145 cells resulted in reduced ZO-1 protein levels, while downregulation of talin was associated with increased ZO-1 (Fig. 3, panels E, F).

**Figure 4:**
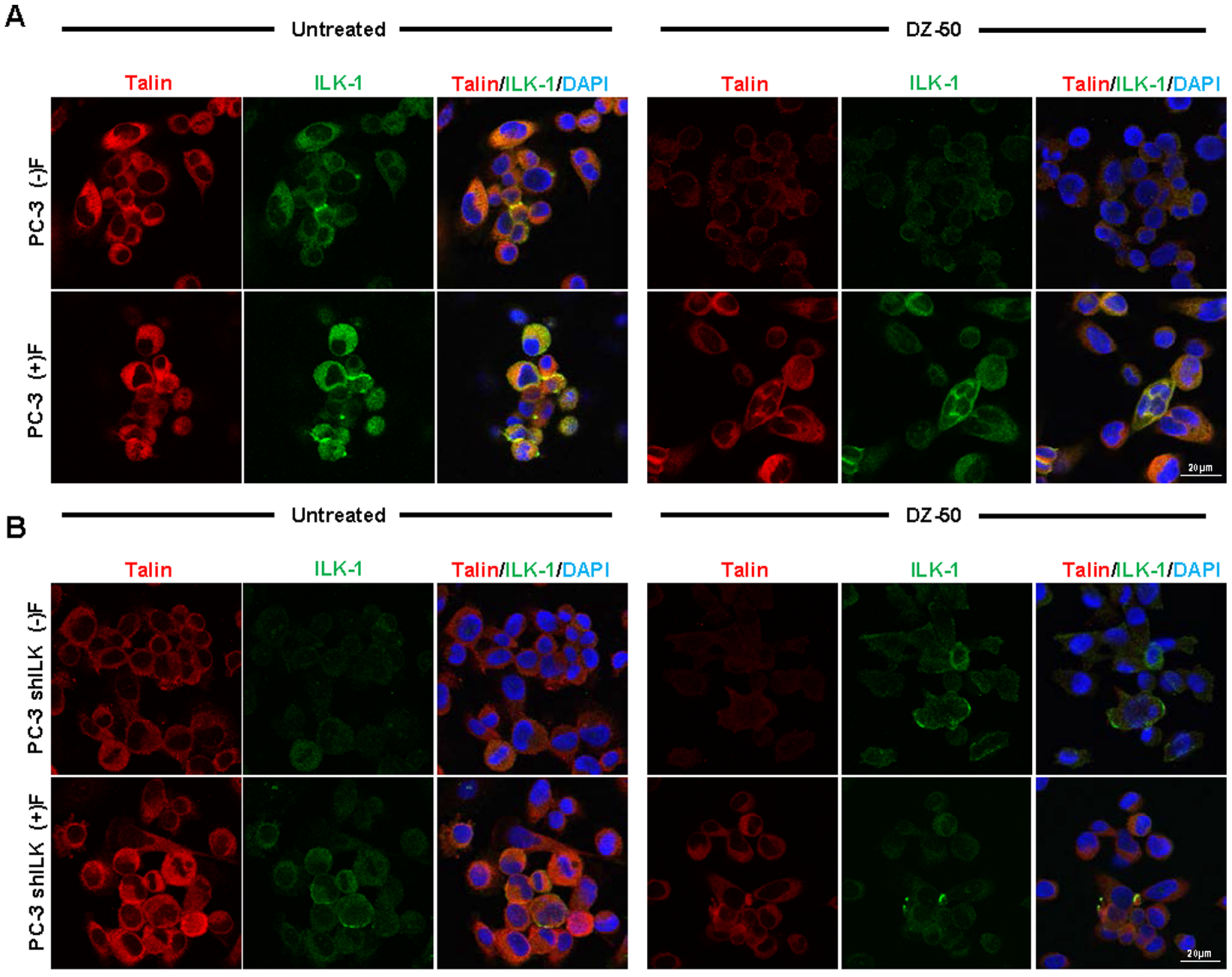
Disruption of focal adhesions in prostate cancer cells by DZ-50. PC-3 prostate cancer cells and PC-3 shILK cells harboring loss of ILK (panels A and B, respectively) were treated with DZ-50 (12 hrs, 5 µM) in the absence or presence of fibronectin-ECM. Fluorescent images reveal the co-localization of focal adhesion regulators talin (red) and ILK (green) to be disrupted by DZ-50 treatment, compared to untreated controls. DAPI (blue) is used for nuclear detection. Silencing ILK expression leads to reduced detection of its primary upstream partner, talin and subsequent disruption of focal adhesions (Panel B), relative to parental PC-3 cells (Panel A, composite, focal adhesions identified in yellow). Prostate cancer cells grown on a fibronectin-coated substrate (ECM integrity) stabilize the focal adhesion complex and diminish the targeting ability of DZ-50 on these substrates in both PC-3 parental and PC-shILK cells. Magnification x100.

**Figure 5:**
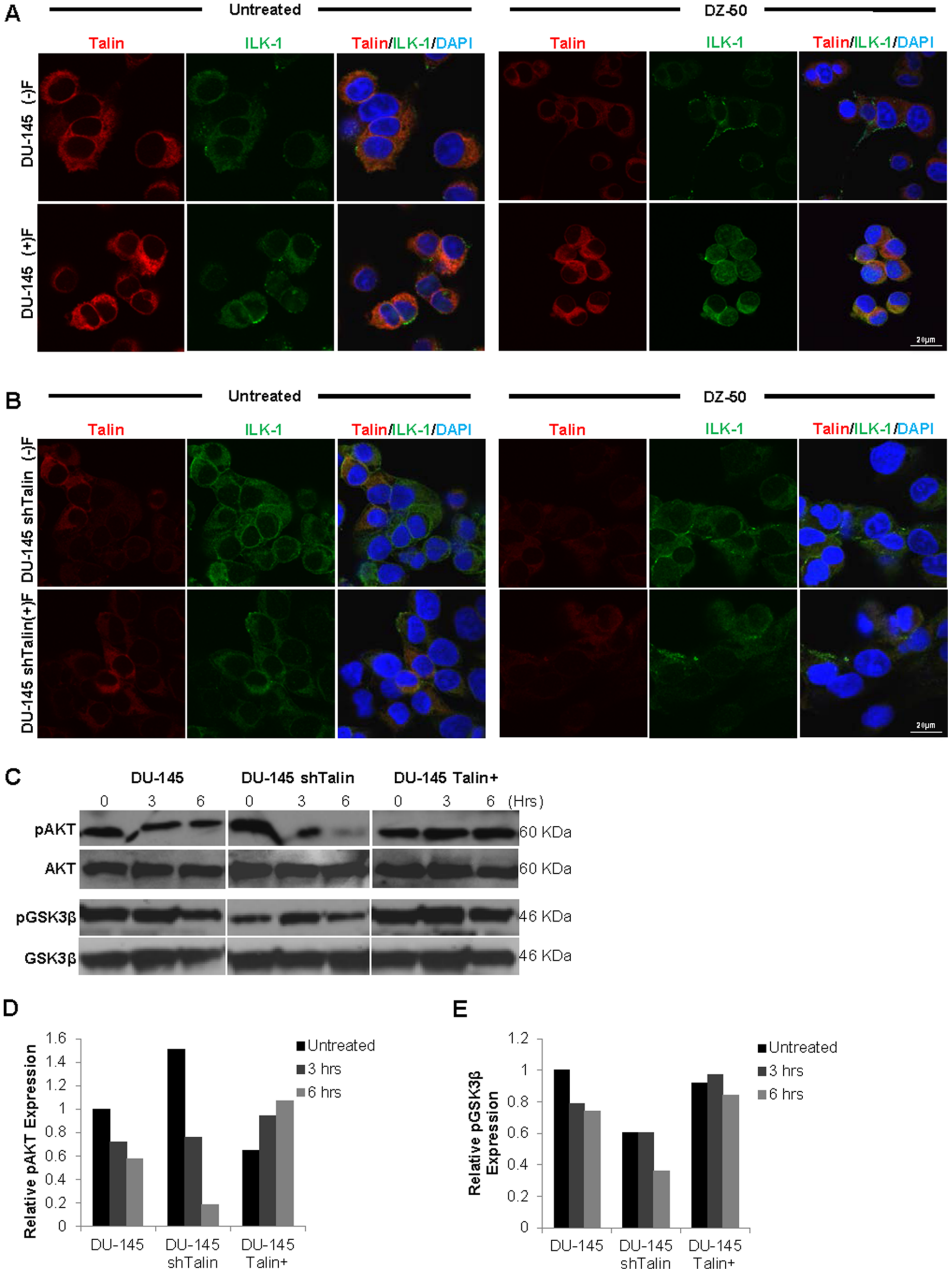
Contribution of Talin-1 to prostate cancer cell anoikis resistance. DU-145 (panel A) and DU-145 shTalin (panel B) were cultured in the absence or presence of fibronectin-coating and treated with DZ-50; cells were subsequently subjected to confocal microscope for detection of talin (red), ILK (green), and focal adhesions (yellow). Nuclei were detected by DAPI staining (Blue). DZ-50 decreased focal adhesion formation through the targeting of talin and ILK. This effects was abrogated by the presence of fibronectin-ECM, which conferred resistance to DZ-50 (Panel A). Loss of talin resulted in reduced co-localization with ILK and disappearance of focal adhesions in DU-145 shTalin cells (Panel B). Magnification x100. Panels C–E, Western blot and quantitative analysis of the time-dependent effect of DZ-50 on downstream cell survival signaling. DZ-50 leads to dephosphorylation of survival signaling effectors AKT (Panel D) and GSK-3β (Panel E). Talin overexpression confers resistance to DZ-50 anoikis effect by sustaining activation/phosphorylation of AKT and GSK-3β signaling.

Fluorescent microscopy was used to examine the effect of DZ-50 on focal adhesion dynamics in the two different human prostate cancer cell lines PC-3 (Fig. 4) and DU-145 (Fig. 5). The results on Figure 4 indicate that in response to DZ-50 (12 hrs), there was a significant decrease in talin (red) and ILK (green) protein expression, compromising the focal adhesion integrity (composite images Fig. 4, panel A; DAPI-blue nuclear staining). To determine the impact of ECM on the cellular response to DZ-50, PC-3 cells were cultured in the presence of fibronectin. The presence of fibronectin facilitated focal adhesion stabilization and sustained expression of talin and ILK, rescuing prostate cancer cells from the anoikis effect of DZ-50 (composite images Fig. 4, panels A and B). Functional loss of ILK in PC-3 cells (Fig. 4, panel B) resulted in FAC instability with concomitant downregulation of talin relative to parental PC-3 cells (Fig. 4, panel A). Treatment of shILK cells with DZ-50 resulted in complete abrogation of FAC signaling, a phenomenon that was partially rescued in the presence of fibronectin.

We subsequently examined the consequences of talin reduction on prostate cancer cell focal adhesion integrity and their sensitivity to DZ-50. Confocal microscopy images shown on Figure 5, reveal that the presence of fibronectin antagonized the anoikis effect of the drug on focal adhesions in DU-145 cells (Fig. 5, panel A) under functional talin levels. Silencing of talin in prostate cancer cells however resulted in the focal adhesion complex formation to be abolished even in the presence of fibronectin (Fig. 5, panel B). There were no so significant differences in focal adhesions in the DU-145shTalin cells.

### DZ-50 Impairs Downstream Intracellular Survival Signaling

**Figure 6:**
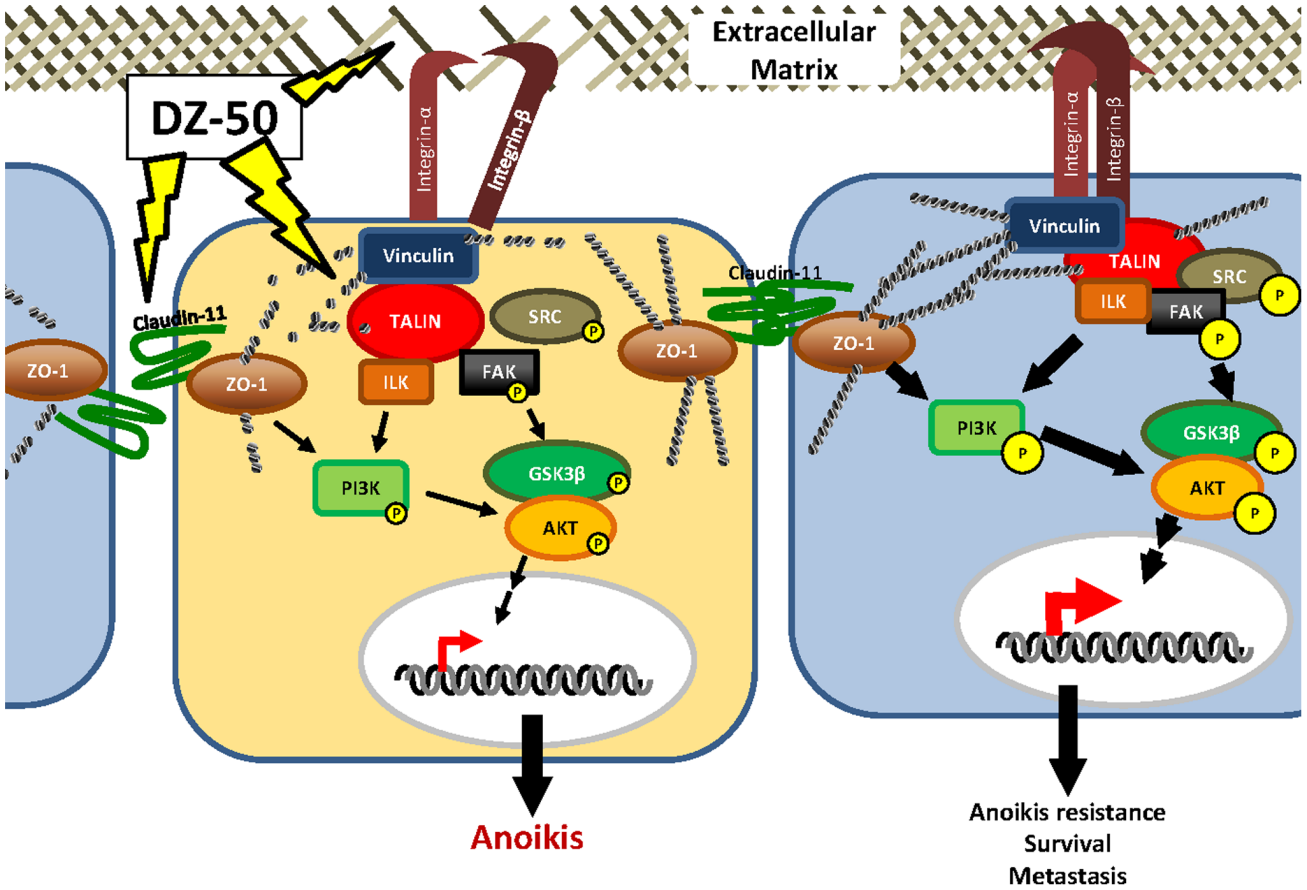
DZ-50 induces anoikis by targeting focal adhesion survival signaling. DZ-50 targets cell-cell interactions (tight junctions) and cell-ECM interactions (focal adhesions) to decrease intracellular survival signals and disrupt actin cytoskeletal integrity to induce anoikis. Stabilization of focal adhesion complex signaling by ECM components and elevated talin, enhances bidirectional integrin signaling, resulting in cellular resistance to anoikis (right). DZ-50 targets extracellular, intercellular and intracellular adhesions and signaling molecules to induce anoikis (left).

To investigate the consequences of DZ-50 on the intracellular signaling downstream of the focal adhesion complex and tight junctions, AKT and GSK3β were profiled as intermediate survival signaling effectors [11], [21]. As shown on Figure 6 targeting of these cellular interactions by DZ-50 results in marked reduction of phosphorylation of both AKT and GSK3β within 3–6 hrs of treatment (Panels B and C). Overexpression of talin in prostate cancer cells induced phosporylation of Akt and GSK-3β, thus leading to enhanced survival and resistance to the action of DZ-50. In contrast, DU-145 cells with reduced talin levels, exhibited decreased Akt and GSK3β phosphorylation (Fig. 6, panel C).

## Discussion

Genome-wide analysis of gene expression in DU-145 human prostate cancer cells revealed downregulation of promising anti-tumor targets by the novel quinazoline derivative DZ-50, including EMT-associated genes (integrin-α6, fibronectin and talin), angiogenesis associated genes (TSP-1), genes associated with intercellular TJs (claudin-11 and 14) as well as serine threonine kinase 31 (TSK31) and insulin growth factor binding protein 3 (IGFBP-3). Confocal microscopy examination confirmed that DZ-50 targets critical proteins involved in the formation of both TJs and focal adhesions, consequently impairing extracellular interactions, actin cytoskeleton integrity and pro-survival intracellular signaling. We previously established the *in vivo* antitumor action of DZ-50 in two human androgen-independent prostate cancer xenografts, PC-3 and DU-145 [7]. Recent evidence suggested that talin confers anoikis resistance in prostate cancer cells towards metastases, via its ability to stabilize focal adhesions and propagate focal adhesion complex signaling through the Akt survival signaling [10]. In the present study, using two different androgen-independent human prostate cancer cell lines and DU-145 and PC-3, as *in vitro* experimental systems, we demonstrated that two distinct intracellular focal adhesion complex components, talin and ILK, are targeted by the lead quinazoline DZ-50 (Fig. 6). Talin overexpression confers insensitivity to DZ-50, while loss of talin reduced tumor cell survival, migration and adhesion, sensitizing prostate cancer cells to the anoikis effect by DZ-50. The phenotypic changes and increased sensitivity to DZ-50 observed in DU-145 cells with low talin expression, and PC-3 cells harboring loss of ILK function, support a regulatory role for these two critical components of the focal adhesion complex in cancer cell anoikis resistance. Figure 6 illustrates a mechanistic schema of DZ-50 mediated anoikis signaling. Tight junctions are located on the apicobasal plasma membrane, forming a selectively permeable barrier essential for fluid and electrolyte balance. Claudins are transmembrane adhesion proteins that span the intercellular space to form homo- or heterodimers on opposing cells [22]. Cytoplasmic tails of claudins interact with actin-stabilizing proteins, zonula occludins (ZO) [23]. Claudin-1 upregulation is associated with colorectal tumor progression via anoikis resistance, evidence linking anoikis to tight junctions impacted by Bcl-2 and AKT survival signaling [24].

Targeting of critical intra- and extracellular focal adhesion components in prostate cancer cells, specifically talin, ILK, integrin-α6 and fibronectin by lead agent DZ-50, lifts anoikis resistance by inhibiting downstream survival signaling by AKT and GSK3β (Fig. 6). Our findings are in accordance with evidence supporting that concomitant activation of FAK and AKT (by transforming growth factor-β1) confers an anoikis-resistant phenotype to myofibroblasts [25]. Moreover, activation of AKT signaling can directly challenge AR activity, functionally implicating this pathway as a contributor to therapeutic resistance to androgen ablation and emergence of CRPC [26]. ECM components facilitate cellular interactions towards stabilization of focal adhesions; our findings suggest that DZ-50 can target fibronectin and integrin expression, disrupting the ECM and ultimately intracellular focal adhesion signaling. Integrins and FAC signaling have been implicated in prostate cancer metastasis to bone through the stabilization of collagen subunits in ECM [27]. Expression profiling α and β integrin subunits revealed upregulation of specific isoforms in prostate tumor metastasis [16], [28], [29], reinforcing a “sniper-targeting” attack against ECM-integrin-FAC to stop metastatic spread.

Epithelial-mesenchymal transition (EMT) confers stem cell properties and leads to acquisition of a migratory, invasive mesenchymal phenotype [30]. A characteristic hallmark of EMT is loss of E-cadherin, causing adherens junction breakdown, which suffices to circumvent anoikis in the tumor microenvironment [31]. Dynamic cycles of EMT-MET can direct the androgen signaling to promote invasive behavior and therapeutic resistance in preclinical models of prostate tumor progression towards CRPC [30], [32], [33]. The role of tight junctions in anoikis and metastasis has not been well understood, despite intense interrogations of the impact of loss of cell-cell interactions in invasive and de-differentiation responses to extracellular stimuli. Overexpression of claudins has been implicated in a variety of tumors including prostate, breast, ovarian and pancreatic cancer [34]. Claudin-1 engages the downstream PI3K/AKT survival pathway, thus contributing to anoikis resistance in colon cancer [24], [35]. Our molecular analysis identified that the lead new quinazoline, DZ-50, disrupts tight junction formation in human prostate cancer cells by downregulating Claudin-11, a critical TJ protein, also contributing to the actin cytoskeleton [36], [37]. Considering the redundant role of maintaining actin cytoskeletal integrity via the AKT pathway shared by tight junctions and focal adhesions, functional interference and potential structural collapse of both cellular-interaction platforms by DZ-50, may contribute to a compounded anti-tumor effect (Fig. 6). Putative targets in cell adherence junctions mediated by E-cadherin in EMT control may also be considered [38].

In summary, the present data identify the anoikis action of the novel lead compound DZ-50 in prostate cancer cells, by disrupting vital cellular interactions navigated by Claudin-11 (TJ) and talin (FAC), in the microenvironment. Elevated Talin may be pharmacologically linked to chemotherapeutic tolerance to drugs that target cell-cell and cell-ECM interactions, pointing to the therapeutic value of DZ-50 in targeting metastatic CRPC. Ongoing clinical trials aim at therapeutic optimization of combined targeting of the AR axis with selective small molecule inhibitors of angiogenesis (tasquinomod, bevacizumab), IGF signaling (cixutumumab), tyrosine kinase signaling (dasatinib, sunitinib, cabozantinib) [2] and proteasome degradation [39]. The anoikis action of DZ-50, enables a pharmacologic platform for development of novel strategies, as well as therapeutic optimization of existing regimes for advanced CRPC [40].
